# Near-infrared quantum cutting luminescence in Pr^3+^/Yb^3+^ doped lead bismuth borate glass

**DOI:** 10.1038/s41598-022-23808-3

**Published:** 2022-11-11

**Authors:** Meruva Seshadri, Ilza T. C. Santos, Maria Jose V. Bell, Jerome Lapointe, Younes Messaddeq, Virgilio Anjos

**Affiliations:** 1 Grupo de Engenharia e Espectroscopia de Materiais, Departamento de Física-ICE, Universidade Federal de Juiz de Fora, Juiz de Fora, MG 36036-900 Brazil; 2 Department of Physics, KG Reddy College of Engineering and Technology, Hyderabad, TS 501504 India; 3 Centre d’Optique, Photonique et Laser, 2375 Rue de la Terrasse, Université Laval, Québec, QC G1V 0A6 Canada; 4 Chemistry Institute, São Paulo State University – UNESP, Rua Francisco Degni 55, Araraquara, SP 14800-900 Brazil

**Keywords:** Materials science, Optics and photonics

## Abstract

In this paper, thermally stable lead-bismuth-borate glasses were doped with 0.5 mol% of Pr^3+^ ions at several concentration levels of Yb^3+^ ions. Structural characterizations were performed via Raman, differential scanning calorimetry, optical absorption and fluorescence spectra. The Judd–Ofelt intensity parameter, ${\Omega }_2$, of Pr^3+^ doped glass was comparatively higher than those from reported ones, which reflects the increase of co-valency and asymmetry of chemical bonds in the local environment of Pr^3+^. Near-infrared emission in 900–2200 nm wavelength range was recorded through 443 nm blue laser pumping. Visible to near-IR quantum cutting and concentration quenching mechanisms were discussed to understand the luminescent behaviour. Intense IR emission ($\sim 1.0\,\upmu {\text {m}})$ features generated by absorbing one visible photon leads to quantum efficiencies close to 128% in Pr^3+^/Yb^3+^ co-doped samples which may improve the solar spectrum absorption and accordingly, increase the efficiency of c-Si solar cells. Emission cross-section, lifetime, figure of merit and gain bandwidth corresponding to Pr^3+^: $^3F_2 \rightarrow ^3H_4$ ($\sim 2.0\,\upmu$m) were comparatively reported suggesting that the glass with molar composition 0.5Pr^3+^/0.1Yb^3+^ might be a potential candidate for $\sim 2.0\,\upmu$m laser operation with low pump threshold.

## Introduction

Quantum cutting (QC) luminescence in materials doped with rare earth (RE) ions is an interesting subject towards the development of fluorescent tubes, plasma display panels and solar cells. In the quantum-cut optical phenomenon, two lower-energy photons are obtained by the energy partition of a high-energy photon. As a consequence, this process opens the possibility of its application in solar cells technology in order to enhance the efficiency of the latter via thermal loss prevention without structural change. Generally, spectra mismatch between solar spectrum and silicon band gap energy are the major gain limiter in c-Si solar cells. The low energy photons can not be absorbed effectively while high-energy photons were not used efficiently. The energy excess is lost in the form of heat during the thermalization of hot carriers^1^. Therefore, the maximum efficiency of a c-Si solar cell is 33% obtained for a band gap of approximately 1.12 eV (1100 nm)^1,2^. Solar efficiencies higher than 33% are also reported for multi-junction solar cells but are limited due to high cost and the requirement of high-quality contacts between the absorber materials while with physical and chemical matching properties^3^.

An alternative approach to enhance the solar cell efficiency is the rare earth (RE) co-doping with luminescent materials known to be efficient QC down-converters. These materials can absorb one UV-visible photon ($< 500$ nm) and generate two IR photons that can be efficiently absorbed by the c-Si solar cells. The most common type of rare earth co-doped systems are Tm$^{3+}$–Yb$^{3+}$^4^, Er$^{3+}$–Yb$^{3+}$^5^, Tb$^{3+}$–Yb$^{3+}$^6^ and Pr$^{3+}$–Yb$^{3+}$^7^ where Tm$^{3+}$, Er$^{3+}$, Tb$^{3+}$ and Pr$^{3+}$ act as absorbing centers. The Yb$^{3+}$ ions act as acceptors and their emitting light energy around 1000 nm is just above the band gap of c-Si solar cell^4^. Usually, in RE co-doped systems, the near-infrared (NIR) quantum cutting process involves the contribution of second-order cooperative energy transfer (CET) and first-order resonant energy transfer (ET) mechanisms. The latter one is more efficient than CET process but there is a possibility of high CET at higher Yb$^{3+}$ ion concentrations^4,7^. Recently, first-order, multiphoton, NIR quantum cutting in Er$^{3+}$, Tm$^{3+}$ ions and Er$^{3+}$/Tm$^{3+}$ co-doped materials have been studied and proved to develop first-order, multi-photon QC germanium (Ge) and Silicon–Germanium (Si–Ge) solar cells (sensitive to 280–1850 nm) with efficiencies exceeding 38%^8^. Pr$^{3+}$–Yb$^{3+}$ co-doped systems have been also studied^9^ due to their efficient optical response in terms of QC phenomenon which can be explained from the emission of Yb$^{3+}$ after resonant excitation of the Pr$^{3+}$: ${^3P_J}$ levels. Van Wijngaarden et al.^10^ theoretically showed first-order resonant mechanism: ${^3P_0} \rightarrow {^1G_4} (Pr^{3+})$ to ${^2F_{5/2}} \rightarrow {^2F_{7/2}} (Yb^{3+})$ or ${^1G_4} \rightarrow {^3H_4} (Pr^{3+})$ to ${^2F_{5/2}} \rightarrow {^2F_{7/2}} (Yb^{3+})$ in ${LiYF_4}$: Pr$^{3+}$/Yb$^{3+}$ crystals. Tanabe et al.^11^ showed also a first order resonance energy transfer mechanism in Pr$^{3+}$/Yb$^{3+}$ co-doped oxyfluoride glass-ceramics. Moreover, Pr$^{3+}$ with its rich spectrum of electronic levels involves a large number of optical transitions in the near- and mid-infrared wavelength range, which have potential uses in amplifiers, remote sensing, tissue welding, micro-surgery, environmental trace gas detection and spectroscopic applications.

In order to achieve efficient near- and mid-infrared emissions, low phonon energy of the host material is required to reduce probabilities of multiphonon relaxations between the electronic levels of RE$^{3+}$ ions. Tellurite^12^, germanate^13^, fluoride^14^ and chalcogenide^15^ glasses doped with RE ions are all promising systems for near- and mid-infrared lasers as they possess low phonon energy. Bi$_2$O$_3$ and PbO based heavy metal oxide (HMO) glasses also possess excellent IR transmission, low phonon energy, high refractive index and good corrosion resistance compared to other conventional oxide glasses. Generally, the B$_2$O$_3$ network former allows wider range of glass forming with heavy metal oxides (PbO, Bi$_2$O$_3$ and WO$_3$) than silicates, phosphates and tellurites. In this way, we have chosen lead-bismuth-borate glass composition, as host matrix, as they may exhibit excellent properties: high density, high refractive index, nonlinear refractive index, broad transmission window and low phonon energy^16–19^. They are also stable, moisture resistant, have relatively low melting temperature and high polarizability (small field strength) of Bi$^{3+}$ and Pb$^{2+}$ cations. Such unique characteristics evince their potential applications in photonics, mechanical sensors, and reflecting windows^20–22^.

In this work, lead-bismuth-borate host matrix were doped with Pr$^{3+}$ and Yb$^{3+}$ ions. While doped lead-borate, bismuth-borate and lead-bismuth-borate glasses have been extensively investigated for nonlinear and magneto-optic applications^23–25^, fewer literature was reported concerning the topics of optical spectroscopy and laser applications. This paper investigates the influence of Yb$^{3+}$ ions on near infrared quantum cutting luminescence ($\sim$ 1.0 & 2.0 $\upmu$m) in Pr$^{3+}$/Yb$^{3+}$ codoped glasses through 443 nm excitation. Moreover, it conducts investigations about radiative excited states lifetimes and about energy transfer mechanisms between Pr and Yb ions.

## Results and discussion

**Figure 1:**
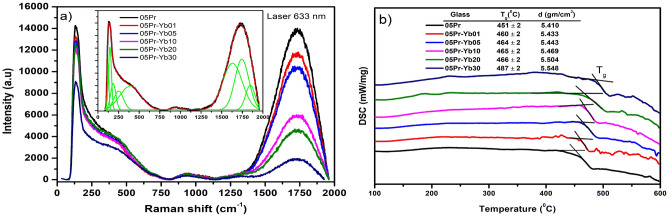
(**a**) Raman and (**b**) DSC spectra of glasses. Inset of (**a**) shows Gaussian fit.

Raman spectra of Pr$^{3+}$/Yb$^{3+}$ doped glasses excited by 633 nm laser and Gaussian fittings of the spectrum, are shown in Fig. 1a. There are ten fitted peaks located at ${\nu }$ ${\sim }$ 121, 145, 184, 250, 392, 955, 1112, 1631, 1744 and 1847 cm$^{-1}$, respectively. The most intense band at frequency ${\sim }$ 200 cm$^{-1}$ is associated to the vibration of heavy metal ions i.e., vibration involving motion of Bi$^{3+}$ cations that are incorporated in the glass network as ${[{\text {BiO}}_3]}$ pyramidal and ${[{\text {BiO}}_6]}$ octahedral units^26,27^. The 250 cm$^{-1}$ can be attributed to the vibrations of Bi–O bonds^27^. The broadband maxima at 392 cm$^{-1}$ is due to Bi–O and Bi–O–Bi vibrations in distorted ${[{\text {BiO}}_6]}$ polyhedra^27^. The bands around 955 and 1112 cm$^{-1}$ arise by the symmetric stretching vibration of B–O bonds in ${[{\text {BO}}_4]}$ tri-, tetra- and penta-borate units^28^. Their intensities constitute an overlapp of B–O bonds with non-bridging oxygens (NBOs) in BO_4_ units. The high frequency bands, 1631, 1744 and 1847 cm$^{-1}$ are associated to the stretching vibrations of the terminal B–O bonds of the pyro-borate units^28^. It is worth to note that the observed decrease of the intense band maxima at low (${\sim }$ 145 cm$^{-1}$) and high (${\sim }$ 1744 cm$^{-1}$) frequencies with the increase of Yb$^{3+}$ content, indicate decrease of Bi$^{3+}$ cations in ${[\text {BiO}_3]}$ and ${[\text {BiO}_6]}$ units, and a decrease number of B–O bonds by the formation of B–O–B linkages in pyro-borate structural units.

Thermal property of the prepared glasses with addition of Yb$^{3+}$ ions are analysed by DSC measurements and are shown in Fig. 1b along with glass transition temperature values. ${T_g}$ is an important parameter to testify the glass thermal stability to resist thermal damage at high pump-power laser intensities for a laser glass. It can be seen that the increase of ${T_g}$ with increase of Yb$^{3+}$ ions from 451 to 487 $^{\circ }$C , indicates the enhancement of rigidity of the glass. This property could be beneficial for stabilization of the glass structure. Density is also a property that reveals the underlying structure of the glass. The density (d) increase of the glasses (see inset data in Fig. 1b) is interrelated with the increase in the packing degree of the glass structure with the increase of Yb$^{3+}$ concentration.

**Figure 2:**
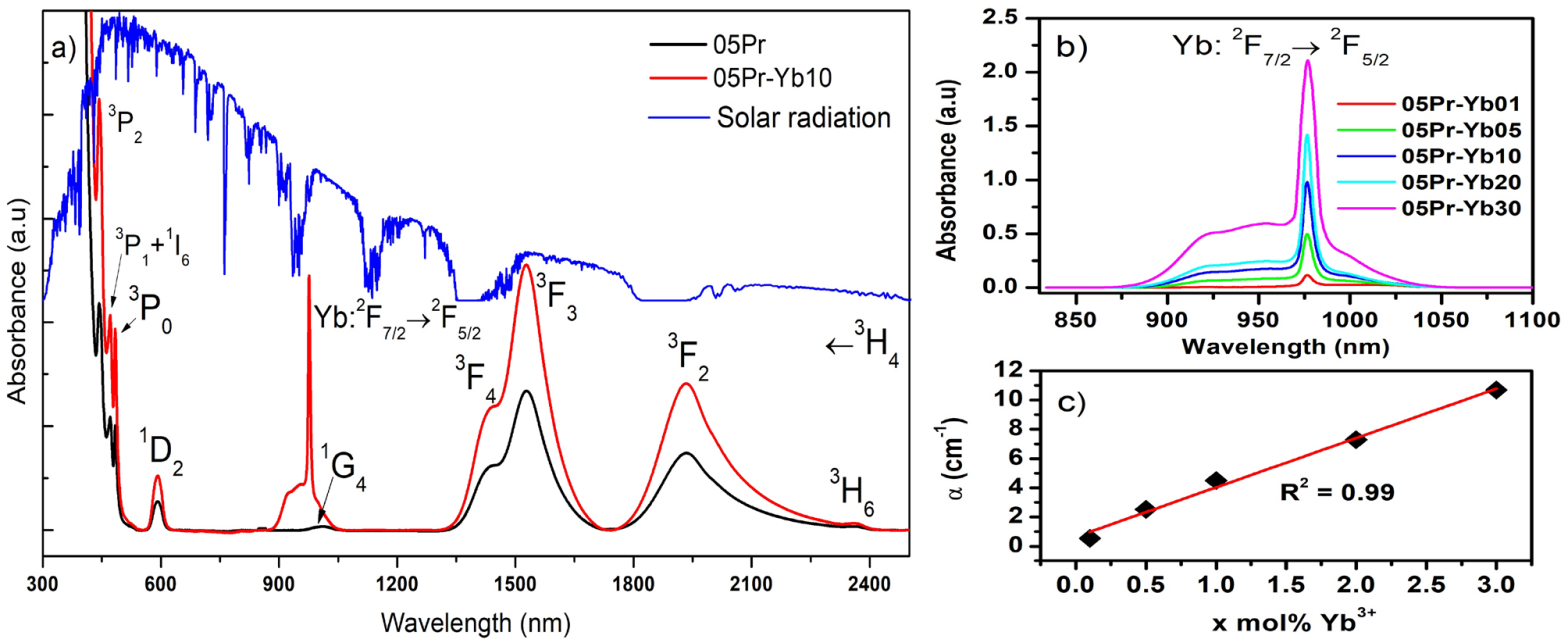
(**a**) Optical absorption spectra of Pr$^{3+}$ singly doped and Pr$^{3+}$/Yb$^{3+}$ co-doped glasses, and sun radiation AM1.5 spectrum; (**b**) absorption intensity of Yb$^{3+}$: ${^2F_{7/2} \rightarrow ^2F_{5/2}}$ transition, and (**c**) integrated intensity of ${^2F_{7/2} \rightarrow ^2F_{5/2}}$ transition as a function of Yb$^{3+}$ ions.

Figure 2a displays the spectra of absorption of Pr$^{3+}$ doped and Pr$^{3+}$/Yb$^{3+}$ co-doped glasses. The absorption bands of Pr$^{3+}$ which correspond to transitions from the ground state ${^3H_4}$ to the excited levels are labeled in Fig. 2a. The absorption band around 1011 nm of Pr$^{3+}$ is weak. Adding Yb$^{3+}$ as co-dopant to Pr$^{3+}$ leads to enhancement of the absorption around the range, 875–1065 nm, as well as, the presence of the strong Ytterbium absorption cross-section at 980 nm related to ${^2F_{7/2} \rightarrow ^2F_{5/2}}$ transition, as labeled in Fig. 2a. This band intensity increases with Yb$^{3+}$ content as shown in Fig. 2b and a linear variation of the absorption coefficient is verified in Fig. 2c, which is an indicative of Yb$^{3+}$ ions solubility in the glass network, revealed by the linear fit (${R^2}$ = 0.99). On the other hand, the absorption of Pr$^{3+}$ in the blue-violet wavelength region is effective to absorb photons, which are not efficiently absorbed by the solar cells. Therefore, co-doping of Pr$^{3+}$ and Yb$^{3+}$ ions are not only applicable to solar cells but also applicable to near-infrared amplifiers due to their unique spectral characteristics.

**Table 1:** Judd–Ofelt intensity (${\Omega _\lambda \times 10^{-20}\,{\text {cm}}^2}$) parameters of different Pr$^{3+}$ doped glasses.

Glass	${\Omega _2}$	${\Omega _4}$	${\Omega _6}$
Lead bismuth borate	1.40	0.30	1.26
Zinc bismuth borate^29^	1.30	3.29	2.13
Lead phosphate^30^	0.34	6.68	5.91
Oxyfluoride^31^	0.13	4.09	6.33
Fluorotellurite^32^	0.98	1.39	13.50
Silicate^33^	0.71	1.33	5.15

Judd–Ofelt (J–O) theory is commonly applied to RE doped glasses to testify the spectroscopic and laser properties such as radiative transition probabilities, radiative lifetime, branching ratios of certain emitting levels of RE ions based on absorption spectrum. Detailed theoretical and calculation method have been well described in previous publications^17,34–37^. Thus, only results for the Lead bismuth borate Pr$^{3+}$ doped glass will be presented. The obtained Judd–Ofelt intensity parameters, ${{\Omega }_{\lambda }}$ (${\lambda }$ = 2, 4 and 6) for several host glasses containing Pr$^{3+}$ ions are reported in Table 1. As it is known, the ${\Omega _2}$ is related to the covalency of RE ions and ligand anions, which reflects the asymmetry of local environment around the RE ions. The covalency of Pr-O bond, in the studied glass, is stronger than those of zinc-bismuth-borate^29^, lead-phosphate^30^, oxyfluoride^31^, fluorotellurite^32^ and silicate^33^ glasses, pointing therefore to stronger asymmetry around the RE ion. ${\Omega _4}$ and ${\Omega _6}$ are associated to the bulk properties like rigidity and viscosity of hosts.

**Figure 3:**
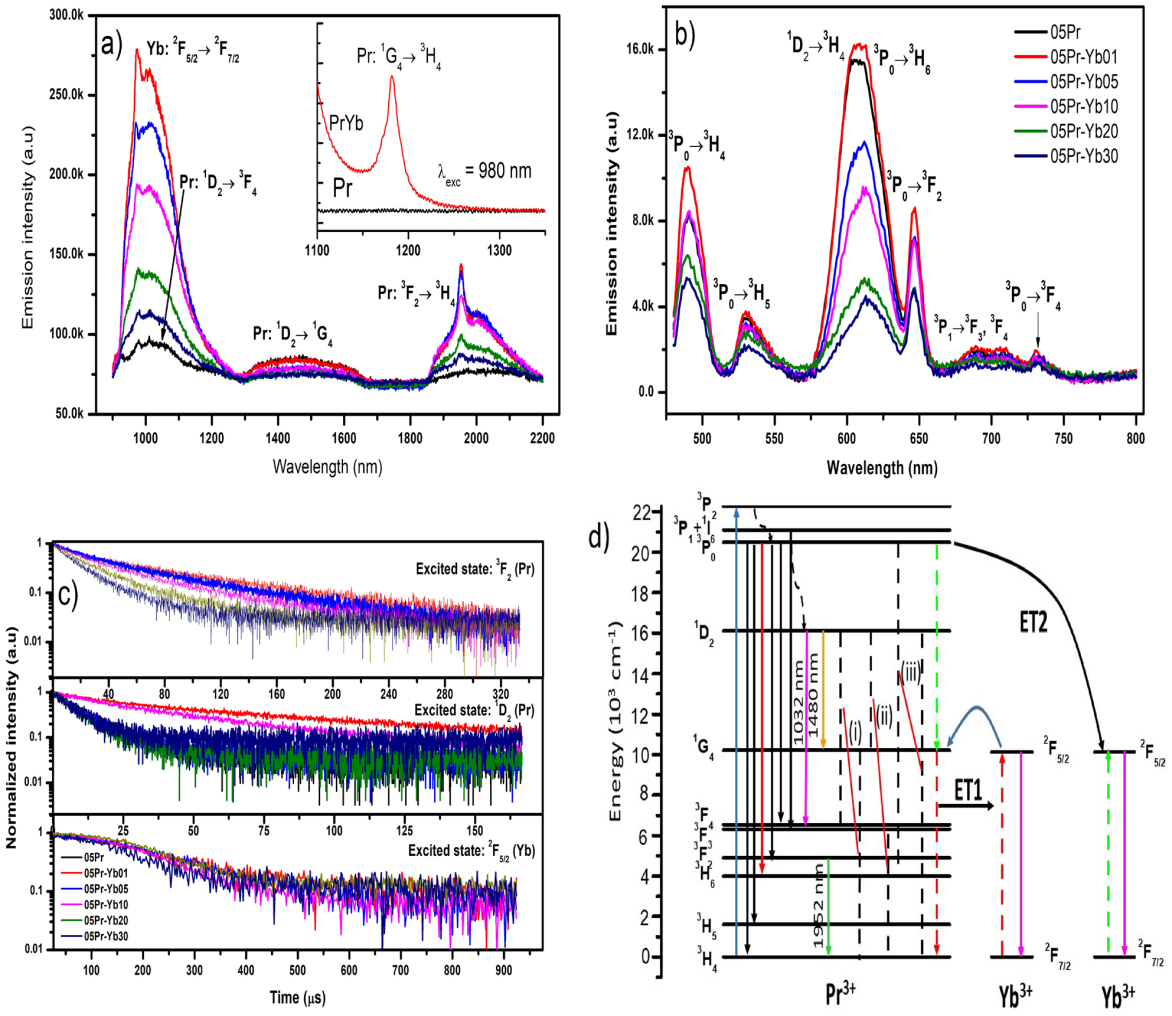
(**a**) NIR emission spectra upon 443 nm excitation and (**b**) visible emission spectra of glasses, (**c**) decay curves of excited levels of Pr$^{3+}$ and Yb$^{3+}$ ions, (**d**) schematic energy level diagram with possible energy transfer between Pr$^{3+}$ and Yb$^{3+}$ ions with cross-relaxation mechanisms. Inset of (**a**) shows NIR emission upon 980 nm excitation.

The most intense absorption peak in the blue region is the result of the excitation of Pr$^{3+}$ ions from ground state, ${^3H_4}$ to excited states, ${^3P_j}$ $({^1I_6})$ (j = 0, 1 and 2), which contribute to NIR and visible emissions (see Fig. 3a, b). In the present work, upon 443 nm excitation, the Pr$^{3+}$ ions are excited to ${^3P_2}$ level and non-radiative decay to ${^3P_0}$ and ${^1D_2}$ levels. Then, radiatively decay to lower levels of Pr$^{3+}$ ions, exhibiting NIR and visible emissions of Pr$^{3+}$ ions (see Fig. 3d). Subsequently, emission corresponding to the transition of Yb$^{3+}$: ${^2F_{5/2} \rightarrow {^2F_{7/2}}}$ emission occurs in Pr$^{3+}$/Yb$^{3+}$ co-doped glasses. In order to feed the ions to Yb$^{3+}$: ${^2F_{5/2}}$ level, there are two possible resonant energy transfer processes involved, ET1: (${^3P_0}$ (Pr$^{3+}$) : ${^2F_{7/2}}$ (Yb$^{3+}$)) ${\rightarrow }$ (${^1G_4}$ (Pr$^{3+}$) : ${^2F_{5/2}}$(Yb$^{3+}$)) and ET2: (${^3P_0}$ (Pr$^{3+}$) ${\rightarrow }$ 2 ${\times }$ (${^2F_{7/2}}$ ${\rightarrow }$ ${^2F_{5/2}}$) (Yb$^{3+}$)) (see Fig. 3d)^38–40^. Most of previous studies point out that the ET1 is more efficient than that of the cooperative energy transfer (ET2).

**Table 2:** Lifetimes of Yb$^{3+}$: ${^2F_{5/2}}$ and Pr$^{3+}$ :${^1D_2}$ & ${^3F_2}$ excited levels as a function of Yb$^{3+}$ in glasses^38,39^.

Glass doped RE$^{3+}$ ions		Lifetime $({\tau })\,({\upmu }\text {s})$			${\eta _{ET}}$	${\eta _{QE}}$
Pr$^{3+}$ (mol%)	Yb$^{3+}$ (mol%)	${^2F_{5/2}}$	${^1D_2}$	${^3F_2}$	%	%
0.5	0	–	11.7	–	–	100
0.5	0.1	195	11.6	72.7	1	101
0.5	0.5	210	10.9	57.1	7	107
0.5	1.0	156	10.3	42.5	12	112
0.5	2.0	144	9.3	29.9	21	121
0.5	3.0	134	8.4	18.5	28	128

As can be seen in the luminescence spectra and energy matching condition of Pr$^{3+}$, Yb$^{3+}$ ions, upon 443 nm excitation, the emission around 900–1300 nm corresponds to the Yb$^{3+}$: ${^2F_{5/2}}$ ${\rightarrow }$ ${^2F_{7/2}}$ (${\sim }$ 980 (1010) nm) transition together with Pr$^{3+}$: ${^1D_2}$ ${\rightarrow }$ ${^3F_4}$ (${\sim }$ 1035 nm) transition. The efficient NIR QC luminescence around 1.0 ${\upmu }$m monotonically decreases with increasing Yb$^{3+}$ ions concentration due to cross-relaxation (CR) process, (${^1D_2}$ ${\rightarrow }$ ${^3F_4}$) : (${^2F_{7/2}}$ ${\rightarrow }$ ${^2F_{5/2}}$), which is predominantly responsible for the luminescence quenching by ions. The decrease of the lifetime of Yb$^{3+}$: ${^2F_{5/2}}$ level (see Table 2) with increase of Yb$^{3+}$ strongly supports the concentration quenching mechanism rather than back transfer of energy mechanism^41,42^. De-excitation of Pr$^{3+}$ ions to ${^1G_4}$ and ${^3F_2}$ + ${^3H_6}$ generates around 1480 nm and 1952 nm simultaneous emission. The ${^1D_2}$ ${\rightarrow }$ ${^1G_4}$ (${\sim }$ 1480 nm) emission is negligibly small and its lifetime decreases from 11.7 to 8.4 ${\upmu }$s due to CR process, (i) (${^1D_2}$ ${\rightarrow }$ ${^3F_{3,4}}$) : (${^3H_4}$ ${\rightarrow }$ ${^1G_4}$) and (ii) (${^1D_2}$ ${\rightarrow }$ ${^1G_4}$) : (${^3H_4}$ ${\rightarrow }$ ${^3F_{3,4}}$)^43^. Similar experimental lifetime of ${^1D_2}$ level was obtained for gallo-germanate glass doped with 0.5 mol% Pr$^{3+}$ ions (12 ${\upmu }$s)^43^. It is worth to note that the multiphonon-relaxation rates from ${^3P_0}$ and ${^1D_2}$ are very small due to an insufficient number of phonons to bridge the energy gap between ${^3P_0}$ ${^1D_2}$ (${\sim }$ 3000 cm$^{-1}$) and ${^1D_2}$ ${^1G_4}$ (${\sim }$ 7000 cm$^{-1}$). Therefore, one can expect that the decrease visible emission intensity of Pr$^{3+}$ via energy transfer followed by CR mechanisms, (ii) and (iii) (${^3P_0}$ ${\rightarrow }$ ${^3H_6}$) : (${^3H_4}$ ${\rightarrow }$ ${^1D_2}$)^40^.

In the present work, the visible emission intensity decreases with increase of Yb$^{3+}$ ions concentration for a fixed concentration of Pr$^{3+}$ at 0.5 mol%. Generally, energy transfer in a pair of ions occurs due to resonant energy levels between donor and acceptor ions, or through phonon assistance. Also, the average distance between Pr and Yb ions is greatly influenced by the concentration of Yb$^{3+}$. Therefore, based on previous assertions, the decrease of visible emission in co-doped systems is likely to be carried out by the CR mechanisms (ii & iii, see Fig. 3d) when the average distance between Pr-Yb is shorter than a critical distance for an efficient energy transfer (${\sim }$10 Å)^44^. The remarkable decrease of visible emission intensity maxima of ${^1D_2} {\rightarrow } {^3H_4}$ (${\sim }$ 604 nm) with respect to ${^3P_0} {\rightarrow } {^3H_6}$ emission (${\sim }$ 612 nm), is due to the competition between the above mentioned CR mechanisms. According to literature^45^, the expected experimental lifetime for ${^1D_2} {\rightarrow } {^3H_4}$ emission is equal to the ${^1D_2} {\rightarrow } {^1G_4}$ (${\sim }$ 1480 nm) emission lifetime values (see Table 2). Moreover, the energy transfer efficiency $({\eta _{ET}})$ between Pr$^{3+}$–Yb$^{3+}$^46–48^ and total quantum efficiency $({\eta _{QE}})$ of ions excited to ${^3P_J}$ levels are important parameters and can be expressed as follows,

1 $$\begin{aligned}{} & {} \eta _{ET} = 1-\frac{\tau _{(Pr.xYb)}}{\tau _{Pr}}, \end{aligned}$$

2 $$\begin{aligned}{} & {} \eta _{QE} = \eta _{Pr}(1-\eta _{ET})+2\eta _{ET}, \end{aligned}$$

where, ${\tau }_{(Pr.xYb)}$ and ${\tau }_{Pr}$ (11.7 ${\upmu }$s) are the average lifetimes with and without Yb$^{3+}$ ions, respectively, and ${\eta }_{Pr}$ is set to be 1^38,39^. Table 2 reports the energy transfer efficiencies for the ${^1D_2}$ ${\rightarrow }$ ${^1G_4}$ transition. The ${\eta }_{ET}$ is increased from 1 % to 28 %, and ${\eta }_{QE}$ is increased from 100 to 128 % with increasing Yb$^{3+}$ ions. The ${\eta }_{QE}$ is an indicative of the ratio increase of emitted photons compared to the absorbed photons in function of the Yb$^{3+}$ concentration.

Figure 3a also shows NIR luminescence around 2.0 ${\upmu }$m in 1850 2200 nm wavelength region, attributed to the Pr$^{3+}$: ${^3F_2}$ ${\rightarrow }$ ${^3H_4}$ transition upon 443 nm excitation. We assume that the ${^3F_2}$ and ${^3H_6}$ multiplets are populated from ${^1G_4}$ levels. As can be seen in Fig. 2a, the ${^3H_4}$ ${\rightarrow }$ ${^1G_4}$ absorption band has low intensity and quite low absorption cross-section which indicates that populating ${^1G_4}$ level by direct excitation is not efficient. Therefore, sensitizing Pr$^{3+}$ with Yb$^{3+}$ is more efficient to populate ${^1G_4}$ level via the emission of Yb$^{3+}$: ${^2F_{5/2}}$ ${\rightarrow }$ ${^2F_{7/2}}$ which nicely overlaps the absorption of Pr$^{3+}$ ${^3H_4}$ ${\rightarrow }$ ${^1G_4}$, indicating that the resonant energy transfer may occur as, (${^2F_{5/2}}$ (Yb$^{3+}$) : ${^3H_4}$ (Pr3+)) ${\rightarrow }$ (${^2F_{7/2}}$(Yb$^{3+}$) : ${^1G_4}$ (Pr$^{3+}$)). Considering the efficient QC in between Pr$^{3+}$ and Yb$^{3+}$ with increasing Yb$^{3+}$ ions, and resonant transfer of energy, the emission of ${^1G_4}$ level should vanish. However, we could not neglect the back transfer of energy from Yb$^{3+}$ which could become more and more efficient inducing an increase of ${^1G_4}$ population. In singly Pr$^{3+}$ doped glass, we could not detect emission from ${^1G_4}$ level, but the Pr$^{3+}$/Yb$^{3+}$ co-doped glass exhibit an emission around 1182 nm which corresponds to Pr$^{3+}$: ${^1G_4}$ ${\rightarrow }$ ${^3H_4}$ transition (see inset of Fig. 3a). Therefore, we believe that the back transfer energy greatly contribute for the observed 2.0 ${\upmu }$m emission in co-doped samples. Unfortunately, the emission intensity of Pr$^{3+}$ (${^3F_2}$ ${\rightarrow }$ ${^3H_4}$) might transfer energy to OH$^-$ groups. The observed fluorescence decay curves for the ${^3F_2}$ excited level are well fitted with single exponential function (Fig. 3c), indicating that there is no significant nonlinear energy transfer between Pr$^{3+}$ ions other than transfer of energy to OH quenching centers. This is confirmed by the decrease lifetime of the excited ${^3F_2}$ level and the measured lifetime written as follows^49^,

3 $$\begin{aligned} \frac{1}{\tau _m} = A_{rad} + W_{mpr} + W_{OH}, \end{aligned}$$

where ${A_{rad}}$ is the radiative decay rate, which is equal to the reciprocal of the decay rate in the absence of OH groups (1/${\tau _0}$). ${W_{mpr}}$ is the multiphonon decay rate, and ${W_{OH}}$ is the energy transfer rate between Pr$^{3+}$ and OH$^-$.

**Table 3:** Emission properties of near 2.0 ${\upmu }$m in RE$^{3+}$ doped glasses.

Glass	${\sigma _e}\,({{\text {cm}}^2})$	${\tau _m}$ (s)	${\sigma _e} \times {\tau _m}$ $({{\text {cm}}^2}\,{{\text {s}}})$
Lead bismuth borate (0.5Pr/0.1Yb)	6.79 ${\times }$ $10^{-19}$	72.71 ${\times }$ $10^{-6}$	4.94 ${\times }$ $10^{-23}$
Tellurite (Ho/Tm)^50^	9.33 ${\times }$ $10^{-20}$	3.29 ${\times }$ $10^{-3}$	3.07 ${\times }$ $10^{-23}$
Germanate (Yb/Tm)^13^	6.90 ${\times }$ $10^{-20}$	1.04 ${\times }$ $10^{-3}$	0.72 ${\times }$ $10^{-23}$
Silicate (Tm)^51^	3.60 ${\times }$ $10^{-20}$	7.91 ${\times }$ $10^{-3}$	2.84 ${\times }$ $10^{-23}$

**Figure 4:**
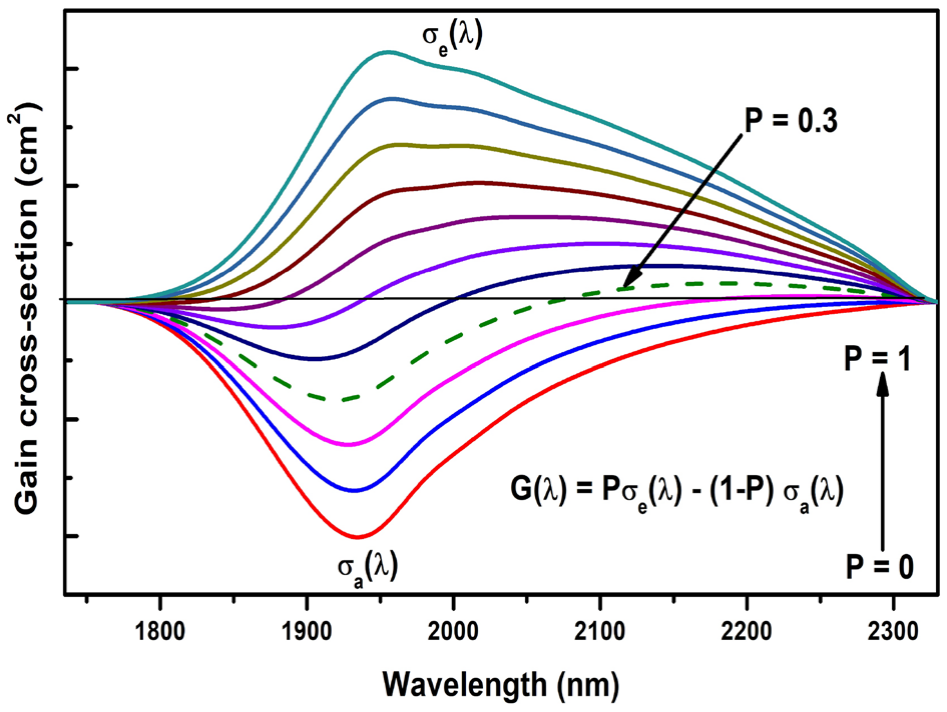
Gain cross-section near 2.0 ${\upmu }$m emission of Pr$^{3+}$.

The gain performance at 1952 nm of the optimized glass can be evaluated through determination of the stimulated emission cross-section^12^,

4 $$\begin{aligned} \sigma _e(\lambda ) = \frac{\lambda _p^4}{8{\pi }cn^2\Delta \lambda _{eff}\tau _m} \end{aligned}$$

where ${\lambda }_p$ is the emission peak wavelength, c is velocity of light, n is refractive index, ${\Delta }_{eff}$ is the effective linewidth and ${\tau }_m$ is the measured lifetime. Table 3 presents important spectroscopic parameters of Pr$^{3+}$ doped for several glasses. The $\sigma _e$ near 2.0 ${\upmu }m$ is one order $(10^{-19})$ higher than those of RE$^{3+}$ doped tellurite^50^, germanate^13^, and silicate^51^ glasses. As it is known, materials which present large stimulated emission cross-section exhibit low threshold and high gain laser operation. In our case, the $\sigma _e$ and figure of merit ($\sigma _e$ ${\times }$ $\tau _m$) are relatively higher than the reported glasses in Table 3, suggesting that 0.5Pr$^{3+}$/0.1Yb$^{3+}$ codoped ${\text {PbO} - \text {Bi}_2\text {O}_3 - \text {B}_2\text {O}_3}$ glass is a promising material for NIR broadband amplifiers. The wavelength dependent gain cross-section can be obtained as a function of population inversion and is written as^12^, G(${\lambda }$) = P${\sigma _e}$(${\lambda }$)-(1-P)${\sigma _a}$ (${\lambda }$), where P is the population inversion of Pr$^{3+}$ ions, absorption and emission cross-sections ${\sigma _a}(\lambda )$ & ${\sigma _e}({\lambda })$ derived from Beer-Lambert and McCumber equations^12^. Figure 4 shows the gain cross-section of Pr$^{3+}$: ${^3F_2}$ ${\rightarrow }$ ${^3H_4}$ transition as a function of population inversion (0–1) varied with an increment of 0.1. It can be seen that the gain becomes positive at P = 0.3 in the range of 2075–2300 nm, which means a low pump threshold of Pr$^{3+}$: ${^3F_2}$ ${\rightarrow }$ ${^3H_4}$ laser operation. Also, the observed positive gain band becomes longer with the increase of P which is a characteristic of a quasi-three level system^51^.

## Conclusions

In summary, Pr$^{3+}$/Yb$^{3+}$ codoped ${\text {PbO} - \text {Bi}_2\text {O}_3 - \text {B}_2\text {O}_3}$ glasses were successfully prepared by melt-quenching technique. The structural, thermal and near-infrared emission properties are investigated. From Raman and DSC results, we found an increase in glass transition temperature, $({T_g})$, with an increase of Yb$^{3+}$ ions concentration which reflects the enhancement of rigidity of the glasses, decrease of Bi$^{3+}$ cations in ${[BiO_3]}$ and ${[BiO_6]}$ units, and decrease of B–O bonds by the formation of B–O–B linkages in pyro-borate structural units. Concerning emissions, we found near-infrared emissions around 980, 1010, 1480 and 1952 nm in the wavelength range 900–2200 nm under 443 nm excitation. The observed concentration quenching is discussed in detail. Quantum effectiveness of the glasses, ${\eta _{QE}}$, has increased from 100 to 128.2% with increasing of Yb$^{3+}$ content which may be used to mimic the solar spectrum aiming its use to enhance the efficiency of c-Si solar cells. The optimized glass (0.5Pr$^{3+}$/0.1Yb$^{3+}$) possess relatively large ${\sim }$ 2.0 ${\upmu }$m $({^3F_2} \rightarrow {^3H_4})$ emission cross-section and high figure of merit implying that this glass can be a promising candidate for ${\sim }$ 2.0 ${\upmu }$m Pr$^{3+}$ laser operation with low pump threshold.

## Methods

Pr$^{3+}$/Yb$^{3+}$ codoped lead-bismuth-borate glasses were prepared by melt-quenching method. The glasses have molar composition of ${(59.5-x) B_2O_3 + 25Bi_2O_3 + 15PbO + 0.5Pr_6O_{11} + xYb_2O_3}$ (x = 0, 0.1, 0.5, 1.0, 2.0 and 3.0). Analytical grade reagents of ${H_3BO_3}$, ${Bi_2O_3}$, *PbO*, ${Pr_6O_{11}}$ and ${Yb_2O_3}$ were used as raw materials, from which nominal batches of 10 g were prepared and mixed in an agate mortar. Then, the mixture was melted in porcelain crucible at 1050 $^{\circ }$C in air for 1 h 30 min and melt was poured into stainless steel moulds. The obtained glass samples were cut and polished for optical characterization.

Differential Scanning Calorimetry was performed with NETZSCH DSC 404F3 with heating rate of 10 $^{\circ }$C/min in order to determine the glass transition ${(T_g)}$ and crystallization temperature ${(T_x)}$ of the glass samples. Raman spectra were recorded with a Renishaw inVia spectrometer coupled with a Leica DM2700 microscope with 633 nm laser excitation. Optical absorption spectra of the glass were recorded on UV-2500 (SHIMADZU) and NIR (BRUKER MPA—Multi Purpose Analyzer) spectrophotometers. Luminescence measurements were performed on Florolog3-iHR HORIBA fluorescence spectrometer upon 443 nm excitation. Density of the glass samples were estimated with distilled water as immersion liquid by Archimedes’ method. All the measurements were conducted at room temperature.

## Data Availability

The data sets generated during and/or analysed during the current study are available from the corresponding author on reasonable request.
