# Publisher Correction: Near-infrared quantum cutting luminescence in Pr^3+^/Yb^3+^ doped lead bismuth borate glass

**DOI:** 10.1038/s41598-023-28611-2

**Published:** 2023-01-25

**Authors:** Meruva Seshadri, Ilza T. C. Santos, Maria Jose V. Bell, Jerome Lapointe, Younes Messaddeq, Virgilio Anjos

**Affiliations:** 1 Grupo de Engenharia e Espectroscopia de Materiais, Departamento de Física-ICE, Universidade Federal de Juiz de Fora, Juiz de Fora, MG 36036-900 Brazil; 2 Department of Physics, KG Reddy College of Engineering and Technology, Hyderabad, TS 501504 India; 3 Centre d’Optique, Photonique et Laser, 2375 Rue de la Terrasse, Université Laval, Québec, QC G1V 0A6 Canada; 4 Chemistry Institute, São Paulo State University – UNESP, Rua Francisco Degni 55, Araraquara, SP 14800-900 Brazil

Correction to: *Scientific Reports* 10.1038/s41598-022-23808-3, published online 11 November 2022

In the original version of this Article Jerome Lapointe and Virgilio Anjos were incorrectly affiliated with ‘Department of Physics, KG Reddy College of Engineering and Technology, Hyderabad, TS 501504, India’. The correct affiliations are listed below.

**Jerome Lapointe:**

Centre d’Optique, Photonique et Laser, 2375 Rue de la Terrasse, Université Laval, Québec, QC G1V 0A6, Canada.

**Virgilio Anjos:**

Grupo de Engenharia e Espectroscopia de Materiais, Departamento de Física‑ICE, Universidade Federal de Juiz de Fora, Juiz de Fora, MG 36036‑900, Brazil.

Centre d’Optique, Photonique et Laser, 2375 Rue de la Terrasse, Université Laval, Québec, QC G1V 0A6, Canada.

The original Article has been corrected.

